# Polymorphism in the Alternative Donor Site of the Cryptic Exon of *LHCGR*: Functional Consequences and Associations with Testosterone Level

**DOI:** 10.1038/srep45699

**Published:** 2017-04-03

**Authors:** Wei Liu, Bing Han, Wenjiao Zhu, Tong Cheng, Mengxia Fan, Jiajun Wu, Ying Yang, Hui Zhu, Jiqiang Si, Qifeng Lyu, Weiran Chai, Shuangxia Zhao, Huaidong Song, Yanping Kuang, Jie Qiao

**Affiliations:** 1 Department of Endocrinology, Shanghai Ninth People’s Hospital, Shanghai Jiao Tong University School of Medicine, Shanghai, China; 2 Research Center for Clinical Medicine, Shanghai Ninth People’s Hospital, Shanghai Jiao Tong University School of Medicine, Shanghai, China; 3 Reproductive Centre, Shanghai Ninth People’s Hospital, Shanghai Jiao Tong University School of Medicine, Shanghai, China

## Abstract

Selective splicing is a feature of luteinizing hormone receptor (LHCGR). A cryptic exon (LHCGR-exon 6A) was found to be derived from alternative splicing in intron 6 of the *LHCGR* gene, which including two transcripts LHCGR-exon 6A-long and LHCGR-exon 6A-short. We addressed the functional consequences of SNP rs68073206, located at the +5 position of an alternative 5′ splice donor site, and observed its association with male infertility in the subjects with azoospermia, oligoasthenozoospermia and normozoospermia. The translation product of splicing variant LHCGR-exon 6A was expressed in the cytoplasm and exhibited no affinity with [^**125**^I]-hCG. No dominant negative effect was observed in cells co-expressed with LHCGR-exon 6A and wild-type LHCGR. The long transcript (LHCGR-exon 6A-long) was significantly elevated in the granulosa cells with G/G genotypes, which could be reproduced *in vitro* by mini-gene construct transfection. Genotyping analysis showed no association between rs68073206 and male infertility. However, this polymorphism was significantly associated with testosterone levels in normozoospermic subjects (n = 210). In conclusion, SNP rs68073206 in the splicing site of the cryptic exon 6A of the *LHCGR* gene affect the splicing pattern in the gene, which may play a role in the modulation of the LHCGR sensitivity in the gonads.

Luteinizing hormone receptor (LHCGR, NM_000233.3) belongs to a subfamily of G protein-coupled receptors (GPCRs) that are responsible for transducing extracellular signals by activating the G protein cascade^1^,^2^. In women, LHCGR signaling plays an essential role in reproduction through the transduction of the signal of the mid-cycle LH surge, leading to ovulation and the subsequent maintenance of progesterone production by the corpus luteum. During pregnancy, human chorionic gonadotropin (hCG), as the second ligand for LHCGR, plays an important role in sustaining progesterone synthesis^3^,^4^. In male fetuses, hCG exerts its effects, which inducing fetal Leydig cell differentiation and testosterone production, during early embryogenesis^5^.

The *LHCGR* gene is located on human chromosome 2p21, and contains 11 exons. The first 10 exons encode the extracellular domain, while the last exon encodes a small portion of the extracellular domain, the transmembrane domain and the cytoplasmic C-terminal domain^1^,^2^,^5^. Selective splicing has proved to be a feature of the glycoprotein receptors, including TSHR and FSHR^6^,^7^. Some *LHCGR* splice variants have also been described in humans and other species, which have been caused by alternative splicing and exon skipping^6^,^8^,^9^,^10^. A cryptic exon which derived from potential splicing sites in intron 6 was identified, resulting in intron retention and producing a cryptic exon—exon 6A^11^. It is noteworthy that 2.7 kbp long genomic region between exons 6 and 7 only present in primates and humans^11^. Two alternative splicing donor sites (GT) have been identified, which, together with the 3′ acceptor site (AG), give rise to a 159 bp (short) or a 207 bp (long) internal exon. In addition, a 3′ polyadenylation signal (AATAAA) was identified and, in cooperation with the 3′ splice acceptor site, yields a terminal exon. Therefore, exon 6A can be spliced into the mature transcripts as a terminal or internal exon. The presence of exon 6A gives rise to at least three splicing variants: without exon6A, with short exon 6A (exon 6A-short) and with long exon 6A (exon 6A-long) (Fig. 1A,B). Kossack *et al*.^11^ demonstrated that transcripts including exon 6A were physiologically highly expressed in human testis and granulosa cells. Moreover, three mutations in exon 6A of the *LHCGR* gene were identified, which could affect the splicing pattern of the gene, leading to down-regulation of the full-length LHCGR.

The polymorphisms of *LHCGR* have been reported to be associated with breast cancer, testicular germ cell cancer, maldescended testes and male infertility^12^,^13^,^14^. Chen *et al*.^15^ found evidence of associations between PCOS and *LHCGR* gene loci by conducting a genome-wide association study (GWAS) of PCOS in Han Chinese women. However, the physiological role of single nucleotide polymorphisms (SNPs) in the cryptic exon and the function of alternatively spliced isoforms derived from exon 6A of *LHCGR* gene remain unclear. Therefore, in this study, we aimed to investigate the function of SNP near the splicing donor site of exon 6A as well as its association with male infertility.

## Materials and Methods

### Genotyping

Genomic DNA was prepared from the peripheral leukocytes of 162 normal subjects (101 male, 62 female), male subjects with azoospermia (n = 133), oligoasthenozoospermia (OAT, n = 138) and normozoospermic (n = 210) using a TIANamp Genomic DNA Kit (Tiangen Biotech, Beijing, China). Semen examination of the patients was performed according to the standardized method of the World Health Organization (WHO)^16^. The experimental protocols were approved by the ethics committee of Shanghai Ninth People’s Hospital affiliated to Shanghai Jiaotong University School of Medicine. Written informed consent was obtained from all participants and the methods were carried out in accordance with the approved guidelines.

SNPs surrounding the region of exon 6A in 162 normal subjects were amplified by PCR using the following primers: LHCGR-in6A-For:5′-TGAGGGTTAGGATTCTTCTCG-3′ and LHCGR-in6A-Rev:5′-TCTTTGAATTCAGGGTGCTCGG-3′. After treatment with shrimp alkaline phosphatase, the PCR products were sequenced in an ABI 3730 DNA sequencer (Applied Biosystems PerkinElmer). The rs68073206 polymorphism was genotyped in subjects with azoospermia, OAT and normozoospermia, using the Custom TaqMan^®^ SNP Genotyping Assay (Life Technologies, USA) on an Applied Biosystems viiA7 Fast Real-time PCR System. Five microliters of PCRs containing 1 μl of DNA (10 ng/μl), 0.125 μl of TaqMan Genotyping probes (Lot number: P130714–005D07), 2.5 μl of TaqMan Genotyping master mix, 0.05 μl of ROX reference dye and 1.375 μl of deionized water were performed using 384-well plates.

### Semen Analysis

The semen samples were collected after 2–7 days of abstinence and were processed within 30 minutes of collection according to the WHO Guidelines, 5th edition^16^. The volume of the semen (mL), sperm concentration (millions/mL), percentages of motile sperm and sperm with normal morphology, and the total sperm count were analyzed in accordance with World Health Organization criteria. Azoospermia (n = 133) was confirmed if no sperm were detected after being centrifuged, concentrated and evaluated again. OAT (n = 138) was defined as a sperm cell count less than 15 × 10^6^ cells/ml in seminal liquid and/or total motility less than 10%. The inclusion criteria for the controls (n = 210) among infertile couples were a sperm concentration ≥15 million/ml, a progressive sperm motility ≥32%, and a normal morphology ≥4%.

### Plasmid construction

The full-length LHCGR cDNA in the pCDNA3.0 plasmid was generously provided by Dr. Aaron J.W. Hsueh. pCDNA3.1-LHCGR, pCDNA3.1-LHCGR-exon 6A, pEGFP-LHCGR and pEGFP-LHCGR-exon 6A were constructed. pCDNA3.1-LHCGR was constructed with a His-Tag, while pCDNA3.1-LHCGR-exon 6A was constructed with 3FLAG-Tag. The pTarget expression plasmid, containing exon 6, exon 6A, exon 7 and 3325 bp of intron 6 of the *LHCGR* gene, was generously provided by Dr. Jӧrg Gromoll. This pTarget LHCGR 6–7 plasmid was used to create the +5 spicing site mutation by site-directed mutagenesis. The primers used to change the nucleotide at the rs68073206 position from T to G (letter in bold) were: LHCGR 6A +5FOR:5′- CTTTGTGTAGATGTAA**G**TTTACATGTA-3′and LHCGR 6A +5REV:5′-**C**TTACATCTACACAAAGGTAAAAGTAC-3′.

### Receptor-binding assay

Receptor-binding assays were performed in both intact cells and detergent-solubilized extracts, as described previously^17^,^18^. Intact cells (2 × 10^**6**^) and detergent-solubilized receptors were incubated overnight at room temperature or at 4 °C, respectively, with increasing amounts of unlabeled hCG (0.1–100 ng/tube) in the presence of [^**125**^I]-hCG (70,000 cpm/0.5 ng, Perkin-Elmer, MA, USA). Non-specific binding was determined in samples containing excess unlabeled hCG (100 IU, Sigma, St. Louis, MO, USA). The experiments were performed in triplicate, and representative data were analyzed using the nonlinear regression curve-fitting computer program GraphPad Prism, version 5.0 (GraphPad SoftwareInc., San Diego, CA, USA).

### Transfection, and Co-immunoprecipitation

293T cells were cultured in 6-well plates to 60% confluence and were cotransfected with different amounts of LHCGR-WT and LHCGR-exon6A-3FLAG using Lipofectamine 2000 (Invitrogen). Forty hours after transfection, cells were harvested, co-immunoprecipitation was performed using a FLAG Immunoprecipitation Kit (Sigma Chemical Co., St. Louis, MO, USA), and 6x-His Tag Monoclonal Antibody (Invitrogen) used for immunoblotting.

### Subcellular localization analysis and immunofluorescence (IFC)

293T cells grown on culture dishes (Nest, Wuxi, China) were transfected with 3.2 μg pEGFP-LHCGR-WT and pCDNA3.1-LHCGR-exon 6A respectively, or co-transfected with pEGFP-LHCGR-WT and pCDNA3.1-LHCGR-exon 6A at different ratios (1:1, 1:2, 1:5) using Lipofectamine 2000. Forty hours after transfection, cells were fixed by 4% paraformaldehyde. Then immunofluorescence was performed as previously described^17^.

### Granulosa cell separation and real-time PCR

With informed consent, follicular fluid was collected from women undergoing transvaginal oocyte retrieval for *in vitro* fertilization, after ovarian stimulation using a standard procedure^10^,^19^. Granulosa cells were obtained from follicular aspirates after the removal of oocytes. Individual follicles were not distinguished, and all of the follicular fluid from the same individual was pooled and centrifuged at 1500 rpm for 10 min. The cells were resuspended in PBS, layered over a 45% Percoll/culture medium mixture, and centrifuged at 1200 rpm for 30 min to pellet the blood cells. The granulosa cells, visible in the interface, were collected by pipette and washed three times in PBS. RNA and DNA were extracted with TRIzol reagent (Invitrogen), according to the manufacturer’s protocol. Real-time PCR was performed with HotStarTaq DNA Polymerase (Qiagen), using the primers and probes described in ref. 12.

### Transfection and real-time PCR

293T cells were cultured in 6-well plates to 60% confluence and were transfected with 0.4 μg of DNA per well using Lipofectamine 2000 (Invitrogen). Forty-eight hours after transfection, the cells were harvested, and total RNA was extracted with TRIzol reagent (Invitrogen), according to the manufacturer’s protocol. cDNA was synthesized using SuperScript^®^ III Reverse Transcriptase (Invitrogen), following the manufacturer’s instructions. RT-PCR was performed using Taq enzyme (Lifefeng Biotechnology, Shanghai, China), and the PCR products were visualized on a 2% agarose gel.

### Hormone data analyses

Peripheral blood samples were collected between 8 a.m. and 10 a.m., and the serum was stored at −20 °C until analysis. Follicle-stimulating hormone (FSH), luteinizing hormone (LH) and testosterone (T) were measured by chemiluminescent microparticle immunoassay (CMIA) on an Abbott architect plus system (Abbott Diagnostics, Abbott Park, IL, USA). The intra- and total-assay CVs were less than 4% and 7.5%, respectively. The androgen sensitivity index (ASI) was defined as the product of the T and LH values.

### Statistical analysis

The statistical analysis of expression data of real-time PCR was performed using one-way ANOVA. The Hardy-Weinberg equilibrium (HWE) was tested in all of the samples using P-link software. The association analysis of rs68073206 was performed in male subjects with azoospermia, OAT and normozoospermia, using the Cochran–Armitage trend test by P-link software. The linkage disequilibrium (LD) block of rs68073206, rs4490239 and rs4637173 was analyzed by Haploview software version 4.2. To compare hormone levels among the normozoospermic subjects with three genotypes (T/T, T/G, G/G of rs68073206), independent sample T-test was applied. SPSS 20.0 statistical software package was used to analyze the data and GraphPad Prism6.0 was used to generate the graphs. The level of statistical significance was considered to be P < 0.05.

## Results

### Genotyping analysis of the SNPs of exon 6A

**Figure 1:**
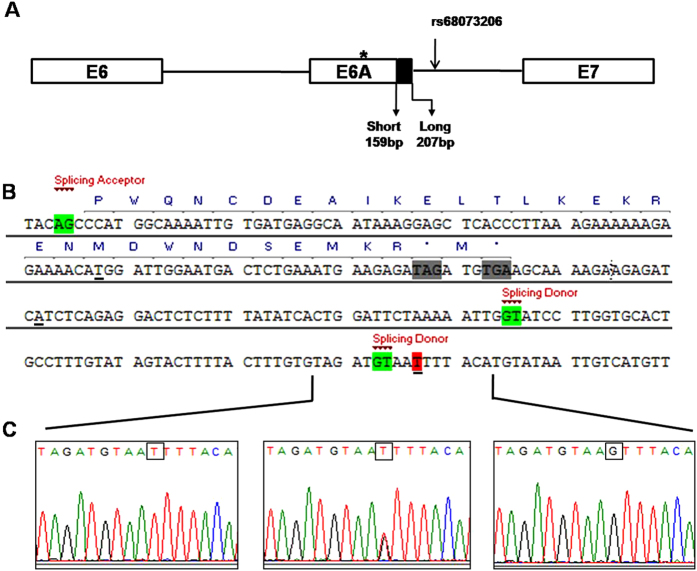
Identification of the alternative splicing sites of exon 6A and the location of rs68073206. (**A**) Schematic representation of the exon 6A and its location in the *LHCGR* gene. The presence of two splicing donor sites give rise to different internal exons—159 and 207 bp. The asterisk indicates the translational stop codon. Rs68073206 is located at +5 of the splice donor site downstream of the long transcripts of 6A. (**B**) DNA nucleotide sequence and putative amino acid sequence of exon 6A. The amino acid sequence is indicated by the blue capital letters above the nucleotide sequence. The splicing donor sites (SD) and acceptor site (SA) are highlighted in green with red notes. The gray shadow indicates the translational stop codons. Three SNPs (rs4637173, rs4490239 and rs68073206) were underlined, while the location of rs68073206 was emphasized in red shadow. (**C**) Sequencing results of rs68073206 with different genotypes (black square).

To explore the cryptic exon 6A of *LHCGR* gene, the exon and the flanking sequence were amplified in 162 normal subjects. Genotyping analysis of the SNPs of the exon 6A was performed by direct sequencing of the PCR product from genomic DNA. Consistent with Kossack *et al*.^11^ reported, 3 SNPs were identified in and around exon 6A. Apart from rs4637173 and rs4490239, which were found to be located in the exonic region of exon 6A, rs68073206 was identified in the intron region following exon6A (Fig. 1A,B). The frequency distributions of these three SNPs are shown in Table 1. Moreover, linkage disequilibrium (LD) analysis in normal subjects showed that rs68073206 was in low LD with rs4490239 and rs4637173 (*r*^2^ = 0) (data no shown). Alternative 5′ splice donor sites (GT), change the 3′ boundary of exon 6A. However, exon 6A was revealed to be an internal exon with characteristics of a splicing signal, giving rise to at least three splice variants through alternative splicing (without exon 6A, exon-6A-short and exon-6A-long). Due to the presence of premature termination codons, variable transcripts with the internal exon 6A, whether 6A-short or 6A-long, produce putatively truncated LHCGR-exon 6A protein with only 209 amino acids, containing of only a part of the extracellular domain and lacking the transmembrane domain (Fig. 1A,B). From the potential splicing site prediction and splicing variants detected by RT-PCR, rs68073206 was proved to be located at +5 of the splice donor site downstream of the long transcripts of 6A (Fig. 1A–C). The particular location of rs68073206, drove our attention to investigate its physical role in the function of alternatively spliced isoforms derived from exon 6A of *LHCGR* gene.

### Receptor-binding assay and subcellular localization analysis of LHCGR-exon 6A

**Figure 2:**
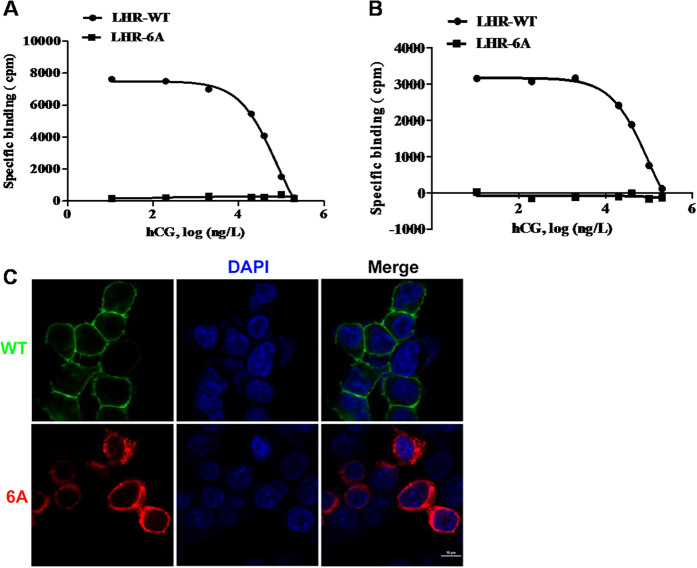
Characterization of ligand binding and subcellular localization of LHCGR-exon 6A. (**A**) 293T cells were transiently transfected with LHCGR-WT or LHCGR-exon 6A. Detergent-solubilized receptors were incubated at 4° C with 1ng of [^**125**^I]-hCG and increasing concentrations of unlabeled hCG. (**B**) Intact cells transfected with LHCGR-WT or LHCGR-exon 6A were incubated at room temperature with constant shaking in the presence of increasing amounts of unlabeled hCG (0.1–l00 ng/tube) and the designated amounts of [^**125**^I]-hCG. (**C**) 293T cells were transfected with GFP-tagged LHCGR-WT plasmids or LHCGR-exon 6A-3flag plasmids. Green (LHCGR-WT) and red (LHCGR-exon 6A-3flag) fluorescence in living cells was determined using a confocal laser microscope with the nucleus stained by DAPI.

We investigated the hormone binding ability of LHCGR-exon 6A with [^**125**^I]-labeled hCG using transfected 293T cells. The specific binding of [^**125**^I]-hCG to detergent-solubilized receptors could be displaced by increasing the concentrations of unlabeled hCG in cells transfected with LHCGR-WT. However, compared with LHCGR-WT, LHCGR-exon 6A exhibited almost no hormone-binding activity, indicating that LHCGR-exon 6A could not bind to its cognate ligand (Fig. 2A). Similar binding patterns were observed in the intact cells transfected with the different constructs (Fig. 2B). To observe the subcellular localization of LHCGR-exon 6A, pEGFP-LHCGR or pCDNA3.1-LHCGR-exon 6A was transfected into 293T cells. The LHCGR-WT was located in the membrane of cells, while the largest portion of LHCGR-exon 6A was present in the cytoplasm (Fig. 2C).

### Analysis the interaction of LHCGR-exon 6A and wild-type LHCGR

**Figure 3:**
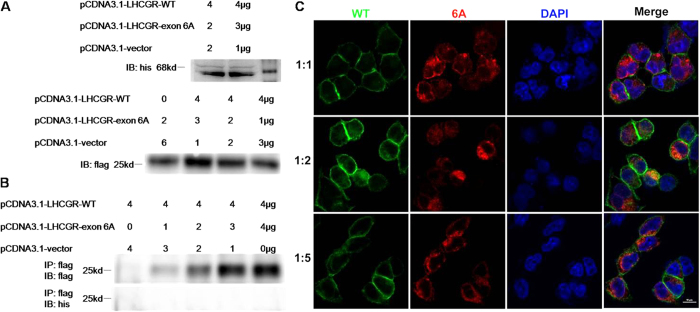
Influence of LHCGR-exon 6A on LHCGR-WT localization. 293T cells were co-transfected with pCDNA3.1-LHCGR-WT and pCDNA3.1-LHCGR-exon 6A-3flag plasmids at different ratios. (**A**) Immunoblot with different antibodies was used to detect the expression of LHCGR-WT and LHCGR-exon 6A. (**B**) Co-immunoprecipitation was performed with FLAG Immunoprecipitation Kit. The interaction between LHCGR-WT and LHCGR-exon 6A was examined with indicated antibodies. (**C**) 293T cells were co-transfected with pEGFP-LHCGR-WT and pCDNA3.1-LHCGR-exon 6A at different ratios (1:1, 1:2 and 1:5). LHCGR-exon 6A was detected by immunofluorescence analysis with anti-FLAG antibody, and observed under cofocal microscopy.

Co-transfection and co-immunoprecipitation was performed to reveal the interaction between LHCGR-exon 6A and wild-type LHCGR. By immunoblotting with anti-FLAG and anti-His antibody, wild-type LHCGR was detected to be a protein of 68 kDa, whereas LHCGR-exon 6A was found to be 25 kDa. However, after co-immunoprecipitation, no interactions of LHCGR-exon 6A and wild-type LHCGR was found, which did not support the dominant negative effect of LHCGR-exon 6A (Fig. 3A,B). When co-transfected with pEGFP-LHCGR-WT and pCDNA3.1-LHCGR-exon 6A at different ratios (1:1, 1:2, 1:5), there was no detectable changes of LHCGR-WT at the cell surface with increasing amounts of LHCGR-exon 6A. These results suggested that the alternative splicing product could not act as a functional receptor nor through a competing mechanism (Fig. 3C).

### Analysis of the relationships between the rs68073206 genotypes and different splicing variant expressions

**Figure 4:**
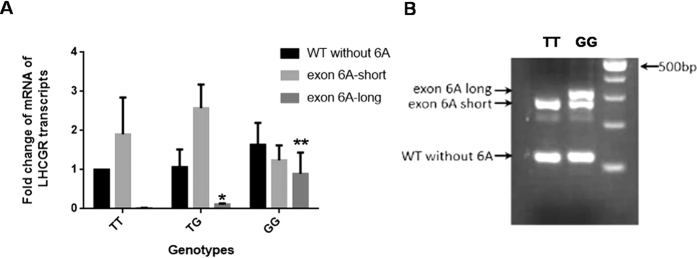
Polymorphism of rs68073206 altered the expression levels of splicing variants around exon 6A. (**A**) The expression levels of wild-type without 6A (WT without exon6A), short splicing variant (exon 6A-short) and long splicing variant (exon 6A-long) were evaluated by real-time PCR in granulose cells from 32 women. By Using GAPDH as internal control, the fold change in the transcripts normalized to GAPDH and relative to the expression levels of WT transcript in the TT genotype, was calculated for each sample using 2^**−ΔΔ**^CT method. The RNA levels of the 6A long transcript were increased significantly in the TG genotype and GG genotype, compared with TT genotype. *P < 0.05 (**B**) RT-PCR showed the expression of different splicing variants in 293T cells transfected with wild-type or T → G mutant mini-gene constructs. The amplification product was clearly visible in the cells transfected with mutant constructs.

The transcripts including exon 6A have been demonstrated to be physiologically highly expressed in human testis and granulosa cells^11^. To reveal the possible effect of the nucleotide change from T to G at the +5 splicing site on the expression of different splicing variants, genotyping analysis and real-time PCR were performed in the granulosa cells of 32 women who underwent IVF treatment. The fold change in the transcripts normalized to GAPDH and relative to the expression levels of WT transcript in the TT genotype, was calculated for each sample. In the granulosa cells, the expression level of the long transcripts was very low – even undetectable– with the T/T genotype, but it was significantly elevated in the T/G and G/G genotypes. It was suggested that the T > G homozygous transversion changed the expression ratio of the different splicing isoforms significantly (Fig. 4A). LHCGR-WT was elevated in the G/G genotype, 1.6 times higher than that in the T/T genotype, and LHCGR-exon 6A-short increased in the T/G genotype but decreased in the G/G genotype without significant difference.

A similar modification was also observed in 293T cells transfected with mini-gene system containing the genomic sequence from exon 6 to exon 7 with wild-type (T) and mutation (G) at +5 of the splicing donor sites. As shown in Fig. 4B, there was a very low – even undetectable – level of 6A-long in the cells transfected with wild-type ptarget-6A plasmid, but the clear amplification product could be observed in the cells transfected with mutant plasmid. It is suggested that the splicing pattern was sustantially changed by the T > G variation of rs68073206.

### Association of rs68073206 with male infertility

The allele frequency and genotype distribution of the rs68073206 polymorphism in the azoospermic (n = 133), oligoasthenoteratozoospermic (n = 138), and normozoospermic subjects (n = 210) are shown in Table 2. The genotyped alleles of all samples were compatible with HWE. Regarding the splicing site polymorphism of rs68073206, the G/G, T/G and T/T genotypes were disclosed to be 13.5%, 45.1%, and 41.4% in patients with azoospermia and 17.4%, 44.2% and 38.4% in patients with OAT, respectively, compared with 15.7%, 47.1% and 37.1% in normozoospermic subjects. No statically significant difference was observed between infertility patients (azoospermia+OAT) and normozoospermic subjects in the dominant model of rs68073206 genotype distribution. Similarly, this splicing site polymorphism showed no genetic association with azoospermia (*P* = 0.70) or with OAT (*P* = 0.85) (Table 2).

### Analysis of the relationship between the rs68073206 genotype and hormone levels in normozoospermic men

**Figure 5:**
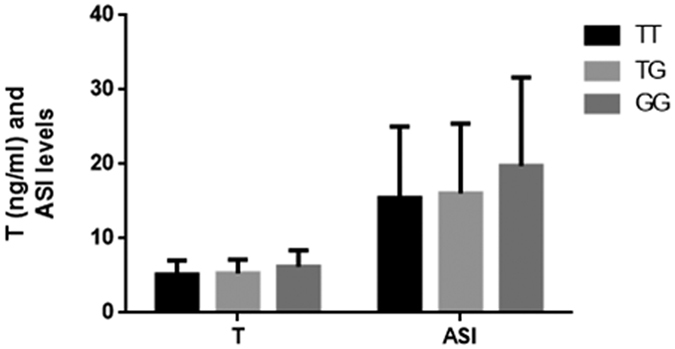
Hormone levels in healthy men among different genotypes of rs68073206. In 210 normozoospermic subjects, T and ASI levels were compared among different genotypes of rs68073206. (*P < 0.05).

The interaction between LH and its receptor is essential for sex steroid secretion in the gonads, which are involved in the process of sex differentiation, puberty and fertility. Because the menstrual cycle and fertility cycle influence the levels of circulating hormones, such as LH and FSH, the hormone levels in women were not analyzed. In the present study, the FSH, LH, T and ASI (LH*T) levels were compared among the 210 normozoospermic individuals with different genotypes. No significant difference in the LH and FSH levels among the T/T, T/G and G/G genotypes was observed in the normozoospermic subjects. However, individuals with the G/G genotype were observed to have significantly higher testosterone levels than those with the T/T genotype (6.20 ± 2.20 vs. 5.16 ± 1.91 ng/ml, *P* = 0.01). Similarly, a significant difference was also observed between the testosterone levels of individuals with G/G genotype and those with T/T+T/G genotype (6.20 ± 2.20 vs. 5.25 ± 1.86 ng/ml, *P* = 0.01). Regarding the ASI, subjects with the G/G genotype showed higher ASI levels than those with the T/T genotype with marginal significance (19.78 ± 11.90 vs. 15.48 ± 9.59, *P* = 0.05). Significant higher ASI levels were also observed in the normozoospermic subjects with G/G genotype compared with those with T/T+T/G genotypes (19.78 ± 11.90 vs. 15.82 ± 9.47, *P* = 0.04), which suggesting the different sensitivity of LHCGR (Table 3, Fig. 5).

## Discussion

Alternative splicing is an important feature of GPCR, which contributes to hormone sensitivity. Exon 6A was presumably derived from a 2.7 kb insertion in intron 6, which is highly conserved in primates, but comparable sequences are completely lacking in mice as well as in other species^11^,^20^. At least three splicing patterns were observed: the full-length receptor transcript without exon 6A, short exon 6A inclusion transcript and long exon 6A inclusion transcript. In our study, we found one of the SNPs located coincidentally at the +5 splicing donor site, with predictable functional consequences. *In vitro* mutagenesis and transfection study demonstrated that T > G transversion created stronger splicing donor signals, leading to significantly increased production of exon 6A inclusion long transcript. The expression ratios of three transcripts were altered remarkably in granulosa cells with G/G genotype, even if the wild-type LHCGR was subtly increased in G/G genotype compared with T/T and T/G genotypes, suggesting the delicate regulation of the function of LHCGR. The genotyping of rs68073206 in azoospermic patients, OAT patients and normozoospermic subjects disclosed a lack of association between rs68073206 and male infertility. However, in the normozoospermic group, significant differences in testosterone levels were detected between subjects with the G/G genotype and patients with the T/T and G/T genotypes.

Although different splicing variants were found to result from the potential splicing sites in intron 6, the translated product was a truncated protein with 209 amino acids because of the existence of two stop codons. Kossack *et al*.^11^,^19^ described four patients with different degrees of Leydig cell hypoplasia (LCH) related to mutations occurring in exon 6A (c.557A > C, c.558G > C and c.580A > G). These mutations increase the number of transcripts containing internal variants of exon 6A, and impair the production of cAMP signal molecules. It is demonstrated that a distinct ratio of these variants is crucial to proper LHCGR function. Therefore, the SNPs located in exon 6A of *LHCGR* changing the ratio of the different transcripts might play a role in modifying or fine tuning LH sensitivity.

A dominant negative effect was demonstrated to be a GPCR regulation mechanism, implemented by a natural splicing isoform. Nakamura *et al*.^20^ demonstrated that the splicing variant of human LH receptor deletion in exon 9 could interact with wild-type LHCGR and attenuate the expression level of the LHCGR receptor. Three alternative splice variants of *LHCGR* in human corpora lutea (CL) and luteinized granulose cells (LGCs) were identified. One of the splice variants encodes a protein lacking the transmembrane and carboxyl terminal domains, and it could regulate the effect of wild-type LHCGR during functional luteolysis^10^,^21^. However, by co-immunoprecipitation and IHC we did not observe the existence of a dominant negative effect between LHCGR-exon 6A and wild-type LHCGR.

The interaction between LH and its receptor is essential for sex steroid secretion in the gonads, which are involved in the process of sex differentiation, puberty and fertility. In a previous study, three SNPs in the exonic region were studied intensively from bioactivity to relationship with disease. The common polymorphic variant (insLQ, rs4539842) in exon 1, resulting from the insertion of the amino acids Leu and Glu in the signal peptide, displayed increased hormone sensitivity and plasma membrane expression compared with the wild-type receptor. It was found to be associated with the shortening of breast cancer disease-free survival, probably by increasing estrogen exposure in female carriers^13^. The two other nonsynonymous SNPs, N291S (rs12470652) and N312S (rs2293275), occur in exon 10, with ethnic diversity in allele frequencies. The N291S LHCGR variant was revealed to alter glycosylation status and to increase receptor sensitivity, but no associations with breast cancer were found, while N312S was more frequent in women with breast cancer than in controls, without significant functional consequence^12^. Furthermore, N312S was also disclosed to be significantly less frequent in men with maldescended testes than in healthy controls, and the difference was confirmed in infertile men with and without maldescensus^14^. A number of splicing variants of GPCR have been demonstrated to play roles in some physical or pathologic processes. Gerasimova *et al*.^22^ revealed four abnormal FSHR splicing mRNA products (deletions of exons 2, 6, and 9 and insertion of a novel exon between exons 8 and 9) in cumulus cells, affecting the extracellular ligand-binding portion of the receptor without causing a frameshift. In particular, splicing variants were found to be associated with low (del ex2) or high (del ex6) responses to controlled ovarian stimulation with FSH.

In our study, SNP rs68073206 at splicing site was found to alter the expression ratios of the splicing variants in either granulosa cells or *in vitro* transfected 293T cells. The long transcript (exon 6A-long) was observed to be significantly elevated in the cells with the G/G genotype, which was very low or even undetectable in T/T and T/G genotypes. Moreover, the expression of wild-type LHCGR was also observed to have the increased trend in the G/G genotype, although no significant difference was detected for limited sample size. Consistent with this finding, testosterone levels were revealed to be elevated in normozoospermic subjects with genotype G/G, compared to those with T/G and T/T genotypes. It has been suggested that a higher expression of the long transcript (exon 6A-long) might be related to a higher level of wild-type LHCGR, resulting in greater sensitivity to LH levels and secretion of testosterone in Leydig cells. However, no association was observed in the polymorphism rs68073206 with azoospermiaor or OAT.

The regulation imposed by alternative splicing product remains unclear. *In vitro* functional studies have suggested that the translated product of the alternative spicing of the cryptic exon is not a functional receptor. In a previous study, Kossack *et al*.^11^,^19^ deduced that the stop codon in exon 6A triggered non-sense mRNA decay (NMD) of transcripts with the inclusion of exon 6A, and the mutations that occurred in exon 6A changed the expression levels of the different transcripts. However, there is no direct evidence supporting that the mRNA of novel transcripts with a portion of intron sequence retention could become the target of NMD. It is conceivable that LHCGR-exon 6A is a non-coding transcript, operating as competing endogenous RNAs (ceRNA) to act as decoys for LHCGR targeting miRNA and to modulate the cellular full-length receptor level^23^,^24^. The recently reported LHCGR binding protein (LRBP), also known as mevalonate kinase, could associate with LHCGR mRNA to form an untranslatable ribonucleoprotein complex and thus inhibit LHCGR mRNA translation^25^,^26^. The interaction between LRBP and LHCGR-exon 6A deserves further investigation.

In conclusion, the SNPs surrounding the potential splicing sites of *LHCGR* could be affected as fine tuning factors in variable gonadotropin sensitivity. Furthermore, larger sample studies should be performed to explore the relationships between special SNP and reproductive disorders and to illustrate the mechanisms that might underlie the associations between alternative splicing and the variable clinical conditions such as infertility.

## Additional Information

**How to cite this article**: Liu, W. *et al*. Polymorphism in the Alternative Donor Site of the Cryptic Exon of *LHCGR*: Functional Consequences and Associations with Testosterone Level. *Sci. Rep.* **7**, 45699; doi: 10.1038/srep45699 (2017).

**Publisher's note:** Springer Nature remains neutral with regard to jurisdictional claims in published maps and institutional affiliations.

## Figures and Tables

**Table 1:** Allele frequencies and genotype distributions of SNPs in exon 6A of *LHCGR* in the normal subjects.

	Genotype frequency			Allele Frequency	
rs68073206	GG	GT	TT	G	T
	16 (9.9%)	80 (49.4%)	66 (40.7%)	35%	65%
rs4637173	AA	AG	GG	A	G
	69 (77.5%)	18 (20.2%)	2 (2.2%)	88%	12%
rs4490239	TT	TC	CC	T	C
	69 (77.5%)	18 (20.2%)	2 (2.2%)	88%	12%

**Table 2:** Association analysis of rs68073206 and the male infertility.

SNP	Allele/genotype	NOR N = 210, n (%)	AZO+OAT N = 271, n (%)	Model	OR (95% CI)	*P*
rs68073206	T	255 (60.7%)	337 (62.2%)	Allellc	1.00 (ref)	0.64
	G	165 (39.3%)	205 (37.8%)		0.94 (0.72–1.22)	
	TT	78 (37.1%)	108 (39.9%)	Geno	1.00 (ref)	0.82
	GT	99 (47.1%)	121 (44.6%)		0.88 (0.60–1.31)	
	GG	33 (15.7%)	42 (15.5%)		0.92 (0.54–1.58)	
	GG	78 (37.1%)	108 (39.9%)	Dominant	1.00 (ref)	0.55
	TT+TG	132 (62.9%)	163 (60.1%)		0.89 (0.62–1.29)	
	TT	33 (15.7%)	42 (15.5%)	Recesive	1.00 (ref)	0.95
	GG+TG	177 (84.3%)	229 (84.5%)		1.02 (0.62–1.67)	
**SNP**	**Allele/genotype**	**NOR N = 210, n (%)**	**AZO N = 133, n (%)**	**Model**	**OR (95% CI)**	***P***
rs68073206	T	255 (60.7%)	170 (63.9%)	Allellc	1.00 (ref)	0.40
	G	165 (39.3%)	96 (36.1%)		0.87 (0.64–1.2)	
	TT	78 (37.1%)	55 (41.4%)	Geno	1.00 (ref)	0.70
	GT	99 (47.1%)	60 (45.1%)		0.86 (0.54–1.38)	
	GG	33 (15.7%)	18 (13.5%)		0.77 (0.4–1.51)	
	GG	78 (37.1%)	55 (41.4%)	Dominant	1.00 (ref)	0.44
	TT+TG	132 (62.9%)	78 (58.6%)		0.84 (0.54–1.31)	
	TT	33 (15.7%)	18 (13.5%)	Recesive	1.00 (ref)	0.58
	GG+TG	177 (84.3%)	115 (86.5%)		1.19 (0.64–2.22)	
**SNP**	**Allele/genotype**	**NOR N = 210, n (%)**	**OAT N = 138, n (%)**	**Model**	**OR (95% CI)**	***P***
rs68073206	T	255 (60.7%)	167 (60.5%)	Allellc	1.00 (ref)	0.96
	G	165 (39.3%)	109 (39.5%)		1.01 (0.74–1.38)	
	TT	78 (37.1%)	53 (38.4%)	Geno	1.00 (ref)	0.85
	GT	99 (47.1%)	61 (44.2%)		0.91 (0.57–1.45)	
	GG	33 (15.7%)	24 (17.4%)		1.07 (0.57–2.01)	
	GG	78 (37.1%)	53 (38.4%)	Dominant	1.00 (ref)	0.81
	TT+TG	132 (62.9%)	85 (61.6%)		0.95 (0.61–1.48)	
	TT	33 (15.7%)	24 (17.4%)	Recesive	1.00 (ref)	0.68
	GG+TG	177 (84.3%)	114 (82.6%)		0.89 (0.5–1.58)	

OR:odds ratio, 95% CI: 95% confidence, Azoospermic (AZO), oligo/asthenospermic (OAT) normozoospermic (NOR).

**Table 3:** Association analyze between rs68073206 genotypes and serum hormone levels of normozoospermic subjects (*P < 0.05).

Hormone	SNP genotype		Mean difference	95% CI	P
	Reference	Variant			
FSH (IU/L)	TT	GG	−0.40	(−1.36:0.56)	0.41
	TT	TG	0.10	(−0.58:0.39)	0.70
	TT	TG+GG	−0.17	(−0.64:0.30)	0.48
	TT+TG	GG	−0.34	(−1.26:1.60)	0.47
LH (IU/L)	TT	GG	−0.16	(−0.71:0.38)	0.57
	TT	TG	−0.03	(−0.43:0.38)	0.89
	TT	TG+GG	−0.06	(−0.45:0.32)	0.75
	TT+TG	GG	−0.15	(−0.63:0.33)	0.56
T (ng/ml)	TT	GG	−1.04	(−1.87:−0.22)	0.01*
	TT	TG	−0.17	(−0.72:0.39)	0.56
	TT	TG+GG	−0.39	(−0.93:0.16)	0.17
	TT+TG	GG	0.95	(−1.67:−0.23)	0.01*
ASI	TT	GG	−4.3	(−8.54:−0.05)	0.05
(LH*T)	TT	TG	−0.60	(−3.43:2.23)	0.68
	TT	TG+GG	−1.52	(−4.33:1.28)	0.29
	TT+TG	GG	−3.97	(−8.40:0.46)	0.04*
